# easyRNASeq: a bioconductor package for processing RNA-Seq data

**DOI:** 10.1093/bioinformatics/bts477

**Published:** 2012-07-30

**Authors:** Nicolas Delhomme, Ismaël Padioleau, Eileen E. Furlong, Lars M. Steinmetz

**Affiliations:** ^1^Genome Biology Computational Support and ^2^Genome Biology Unit, European Molecular Biology Laboratory, Meyerhofstrasse 1, 69117 Heidelberg, Germany

## Abstract

**Motivation:** RNA sequencing is becoming a standard for expression profiling experiments and many tools have been developed in the past few years to analyze RNA-Seq data. Numerous ‘Bioconductor’ packages are available for next-generation sequencing data loading in R, e.g. ShortRead and Rsamtools as well as to perform differential gene expression analyses, e.g. DESeq and edgeR. However, the processing tasks lying in between these require the precise interplay of many Bioconductor packages, e.g. Biostrings, IRanges or external solutions are to be sought.

**Results:** We developed ‘easyRNASeq’, an R package that simplifies the processing of RNA sequencing data, hiding the complex interplay of the required packages behind a single functionality.

**Availability:** The package is implemented in R (as of version 2.15) and is available from Bioconductor (as of version 2.10) at the URL: http://bioconductor.org/packages/release/bioc/html/easyRNASeq.html, where installation and usage instructions can be found.

**Contact:** delhomme@embl.de

## 1 INTRODUCTION

Since the extensive utilization of RNA sequencing for expression profiling (RNA-Seq, Mortazavi *et al.*, 2008), numerous tools have been developed, as part of the R/Bioconductor (Gentleman *et al.*, 2004) project, to load RNA-Seq data in R. The first: ‘ShortRead’ (Morgan *et al.*, 2009) parses manufacturer-specific formats. It gave way to the ‘Rsamtools’ package, as the ‘SAM/BAM’ format (Li *et al.*, 2009) became a *de facto* standard for reporting next-generation sequencing (NGS) alignment data. In parallel, analysis packages were adapted (‘edgeR’, Robinson *et al.*, 2010) or newly developed (‘DESeq’, Anders and Huber, 2010) to accommodate for NGS specificities. Recently, the ‘Bioconductor’ Core Team released several packages to connect these parts of the process: e.g. GenomicRanges, GenomicFeatures; however, combining them appropriately requires a good understanding of their functionalities, and depending on the data, different combinations of these packages have to be used, implying a long learning curve for RNA-Seq neophytes. In parallel to these aspects, the sequencers’ yield increase resulted in the generalization of protocols that allow several samples to be run in a single lane, a process called ‘multiplexing’ (Lefrançois *et al.*, 2009). De-multiplexing the obtained data is a processing step that no R package currently addresses. Here, we describe ‘easyRNASeq’, an R package that eases RNA-Seq processing by combining the necessary packages in a single wrapper that ensures the pertinence of the provided data and information and helps users circumnavigate RNA-Seq processing pitfalls. In addition, it introduces functionalities to handle data produced by recent NGS protocols.

## 2 easyRNASeq

**Fig. 1.**
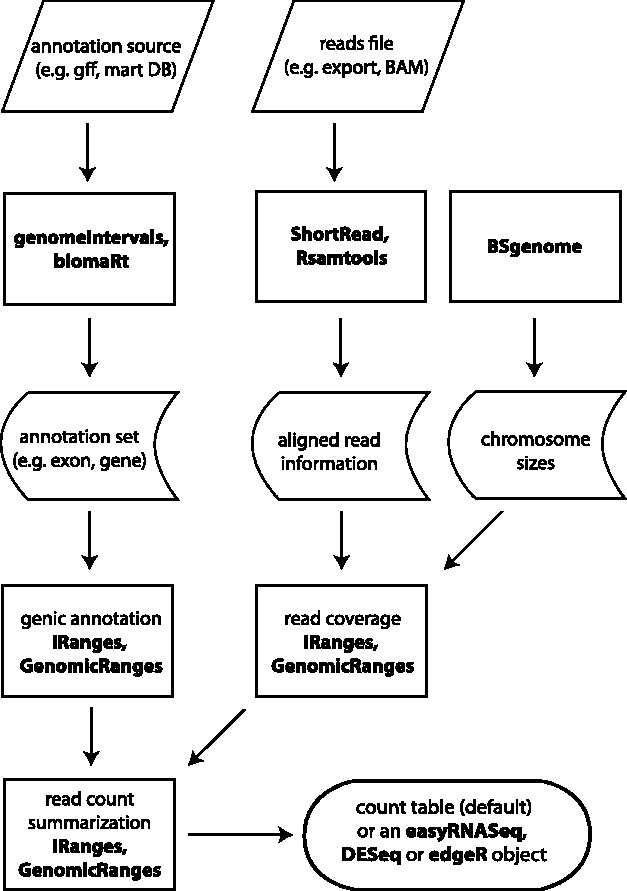
Packages wrapped by easyRNASeq. At every step of the process, easyRNASeq encapsulates and extends lower level package functionalities, finally merging them into a single high-level function.: *easyRNASeq*

The easyRNASeq package combines the following steps: reading in sequenced reads, retrieving annotations, summarizing read counts by the feature of interest, e.g. exon, gene and finally reporting results, normalized or not, in formats suitable for downstream analyses. This is achieved by using and extending many Bioconductor packages functionalities (Fig. 1) and provided to end users as a single function that wraps the entire process.

### 2.1 Reading data

Depending on the alignment format, manufacturer-specific (e.g. Illumina export) or the *de facto* BAM (Binary Alignment/Map) standard, the data are parsed by either ShortRead or Rsamtools, respectively. The coverage is extracted per base pair and divided by the read length—i.e. reads’ coverage proportion are reported per base pair—and stored in an ‘IRanges running length encoding’ (RLE) vector. This approach yields identical results to the common counting reads per se approach, when applied to non-spliced regions as shown in the ‘RNASeqTutorial’ vignette. However, it more accurately assigns reads spanning exon–exon junctions (EEJ), i.e. unlike common methods that arbitrarily select an EEJ side, reads coverage proportion is, here, distributed across the EEJ.

### 2.2 Loading annotations

Genic annotations are retrieved using ‘biomaRt’ (Durinck *et al.*, 2005) or read from ‘General Feature Format 3’ (GFF3) or ‘Gene Transfer Format’ (GTF) files using ‘genomeIntervals’. The annotation set is stored in a ‘RangedData’ object (IRanges). To reduce loading times, ‘RangedData’ or ‘GRangesList’ objects from the R environment or *RData* (rda) files can be used, provided they describe exons, features, transcripts or genes (a ‘feature’ is the representation of a genomic locus, not necessarily genic, e.g. an enhancer).

### 2.3 Counting reads

The reads’ coverage is summarized according to the chosen features: exons, features, transcripts or gene models. Here, a gene model is defined as the set of non-overlapping loci (i.e. synthetic exons) that represent all the possible exons and untranslated regions of a gene. easyRNASeq is not limited to genic summarization only, e.g. promoter ‘features’ can be used to look for eRNAs (Kim *et al.*, 2010).

### 2.4 Output

Four formats are offered: count table (the default), ‘CountDataSet’ (*DESeq* object), ‘DGEList’ (*edgeR* object) and ‘RNAseq’ (*easyRNASeq* object). The count table reports raw counts or ‘reads per kb of feature per million reads’ (RPKM, Mortazavi *et al.*, 2008), if preferred. For *DESeq* and *edgeR*, an object of their respective class is returned. If desired, these would have been subjected to their respective normalization, in which case quality assessment (QA) plots are drawn to evaluate it. Finally, *RNAseq* objects allow different count summarizations to be performed (e.g. by exon, by gene) on the same data without re-processing, a useful feature when first assessing a dataset.

### 2.5 Performance

Fetching annotation using ‘biomaRt’ takes about 10 min from an average network. Generating the gene models takes up to 15 min for large genomes—e.g. Homo sapiens. If these annotations are readily available, processing a 36-bp single-end ‘Illumina’ lane with 100 M reads—a BAM file of 3.2 GB—requires 3 GB RAM and 3 min on an Intel 2.4 GHz CPU.

## 3 DE-MULTIPLEXING

The current data yield allows several samples to be sequenced as a single library, where small nucleotide ‘barcodes’ (4–6 bp) uniquely identify the samples. Resulting sequences must be ‘de-multiplexed’, a functionality introduced by easyRNASeq that splits the result file into sample-specific files. To account for sequencing errors, flexibility in identifying the barcode is necessary and achieved using thresholds based on the Hamming distance (Hamming, 1950). QA plots help validating the chosen thresholds as well as assessing the multiplexing efficacy.

## 4 CONCLUSIONS

This note presents the Bioconductor easyRNASeq package. It introduces a method that effectively hides the complex interplay of numerous Bioconductor packages to the end user. Its output can be formatted for further processing by analysis packages such as DESeq or edgeR. Finally, it contains additional features such as de-multiplexing or support for gapped alignments. Future developments will integrate recent trends such as strand-specific sequencing or differential exon usage detection methods.
